# Evaluation of memory drought stress effects on storage compounds seedlings of cotton (*Gossypium hirsutum*) and in-silico analysis of glutathione reductase

**DOI:** 10.1186/s12870-024-05522-6

**Published:** 2024-09-03

**Authors:** Elham Faghani, Amenehsadat Hashemi, Mina Kazemian, Mohammad Hossein Razzaghi

**Affiliations:** 1 Cotton Research Institute, Agricultural Research, Education and Extension Organization (AREEO), Gorgan, Iran; 2 Agricultural Department, University of Applied Science and Technology, Sari, Mazandaran Iran; 3 Department of Plant Biology, Faculty of Natural Sciences, University of Tabriz, Tabriz, Iran; 4 Agricultural Engineering Research Department, Golestan Agricultural and Natural Resources Research and Education Center, Agricultural Research, Education and Extension Organization (AREEO), Gorgan, Iran

**Keywords:** Cottonseed, Drought, Glutathione reductase, Memory stress

## Abstract

In breeding programs, stress memory in plants can develop drought stress tolerance. Memory stress, as an approach, can keep stress data by activating tolerance mechanisms. This research was conducted to evaluate some physiologically effective mechanisms in inducing memory drought stress in the seeds that were exposed to water stress three times in four treatments including rainfed, 33%, 66%, and 100% of field capacity (FC). After the production of the seeds, the third-generation seeds were placed under different irrigation treatments, seed and seedling traits, starch to carbohydrate ratio in seed, protein concentration and glutathione reductase were investigatied in a factorial format based on a randomized complete block design with three replications. Results showed that percentage of changes from the lowest to the highest value for traits including seed vigor, seed endosperm weight, seed coat weight, accelerated aging, cold test, seedling biomass and seedling length were 25, 37, 65, 65, 55, 77, 55, 65 and 79, respectively and germination uniformity was 3.9 times higher than the lowest amount. According to the deterioration percentage, seed vigor and the percentage of seed germination in cold test data, it can be reported that seed production by 100% FC was not appropriate for rainfed plots. However, considering the the appropriate results in the percentage of germination for a cold test, germination uniformity percentage, and the lowest accelerated aging seeds, seed production under the rainfed conditions with 33% FC watering can be recommended. In-silico analysis was coducted on Glutathione reductase (GR) enzymes in *Gossypium hirsutum*. It is clear that GR has a Redox-active site and NADPH binding, and it interacts with Glutathione S transferase (GST). So, memory drought stress through inducing physiological drought tolerance mechanisms such as starch-to-carbohydrate ratio and GR can determine the suitable pattern for seed production for rainfed and low rainfall regions in a breeding program. Our study thus illustrated that seed reprduction under 33% FC equipped cotton with the tolerance against under draught stress from the seedling stage. This process is done through activating glutathione reductase and balancing the ratio of starch to carbohydrates concentration.

## Introduction

Drought stress, as the most common abiotic stress, causes negative effects on developmental, physiological, biochemical, and molecular traits [10]. It seems that global warmaing plays a significant role in expanding drought stress based on 30 years analysis of average temperature during flowering evaluation temperature data [40]. Sugumar et al. [40]. reported that the phenomenon of climate change affects the number of days and the severity of water deficiency that cotton plants are exposed to. This, consequently, can cause changes of seed reproduction strategies. Being in exposure to a small degree of drought stress in advance can enhance the plant’s adaptability to subsequent stress [45].

Plants have evolved various regulatory mechanisms to cope with the changes in environmental conditions. It is clear that plants, in exposure to drought stress subsequently during a life cycle, can establish different adaptation and tolerant mechanisms [48]. One of these strategies is called stress memory, which causes the plant to have an incremental response when exposed to the subsequent water shortage stresses [15]. Achieving stress memory may be a response at the transcriptional level that is associated with increased transcription and transcript levels of stress response genes that are produced during subsequent stresses on the plant [7].

According to Soriano et al. [39] study, abscisic acid (ABA) may be involved in drought stress memory in a short period; however, epigenomic variations play an essential role in meristem functioning of seedling growth, seed development, and crop yield in the long term [2]. As Moloi et al. [26] indicated, soluble sugar concentration is evaluated as the primary drought tolerance indicator [26]. Moreover, Makonya et al. [24] reported that non-structural carbohydrates (glucose and sucrose) and starch are the primary sources of energy for plant growth, which are used for the allocation of carbon and osmolytes. Also, it is understood that non-structural carbohydrate such as starch was rebuilt after drought stress [16]. Furthermore, restrictions on sharing of starch leads to survival of seedlings under a drought stress condition. Conversely, Glutathione as a substrate or co-factor for several biochemical reactions, not only copes with hormones and redox molecules but also takes part in signal transduction under abiotic stress conditions [27]. In this regard, Wang et al. [44] indicate glutathione reductasse enhances stress resistance in plants, even though many functions of glutathione in plants under environmental stress remain unknown.

It is clear that decrease in seedling dry weight, seed germination percentage and age acceleration in seeds under a drought stress condition are resulted by increase in hydrogen peroxide (H_2_O_2_) and O_2_ in the radical and cotyledon leaves of seedlings [45]. On the other side, glutathione reductase is able to diminish this damage effect on seedlings by increasing ABA and balancing growth regulation compounds [5]. High activity in glutathione reductase is derived from ROS detoxification role in exposure to (H_2_O_2_) and O_2_^−^ accumulation. Glutathione influences the growth of seedlings through its regulating effect on cell division in the root meristemic segments to the extent that this function is important for morphological adaptation to drought stress, which consequently emphasizes memory stress role [18]. Sairam et al. [34]. figured out that increasing both glutathione reductase activity and the amount of glutathione reductase expression causes plants to be more tolerant to oxidative stress [38]. It is hyphosized that by supplying about 30% total water requirement during flowering, seeds will have potential cotton seed through activatiting memorial drought stress.

The current expriment was conducted as an innovative study on cotton to evaluate memory drought stress effects on seed vigor and seed deterioration by evaluating Glutathione reductase activity role in drought stress, in particular in breeding programs for achieving suitable emergence in rainfed farms.

## Material and method

**Fig. 1:**
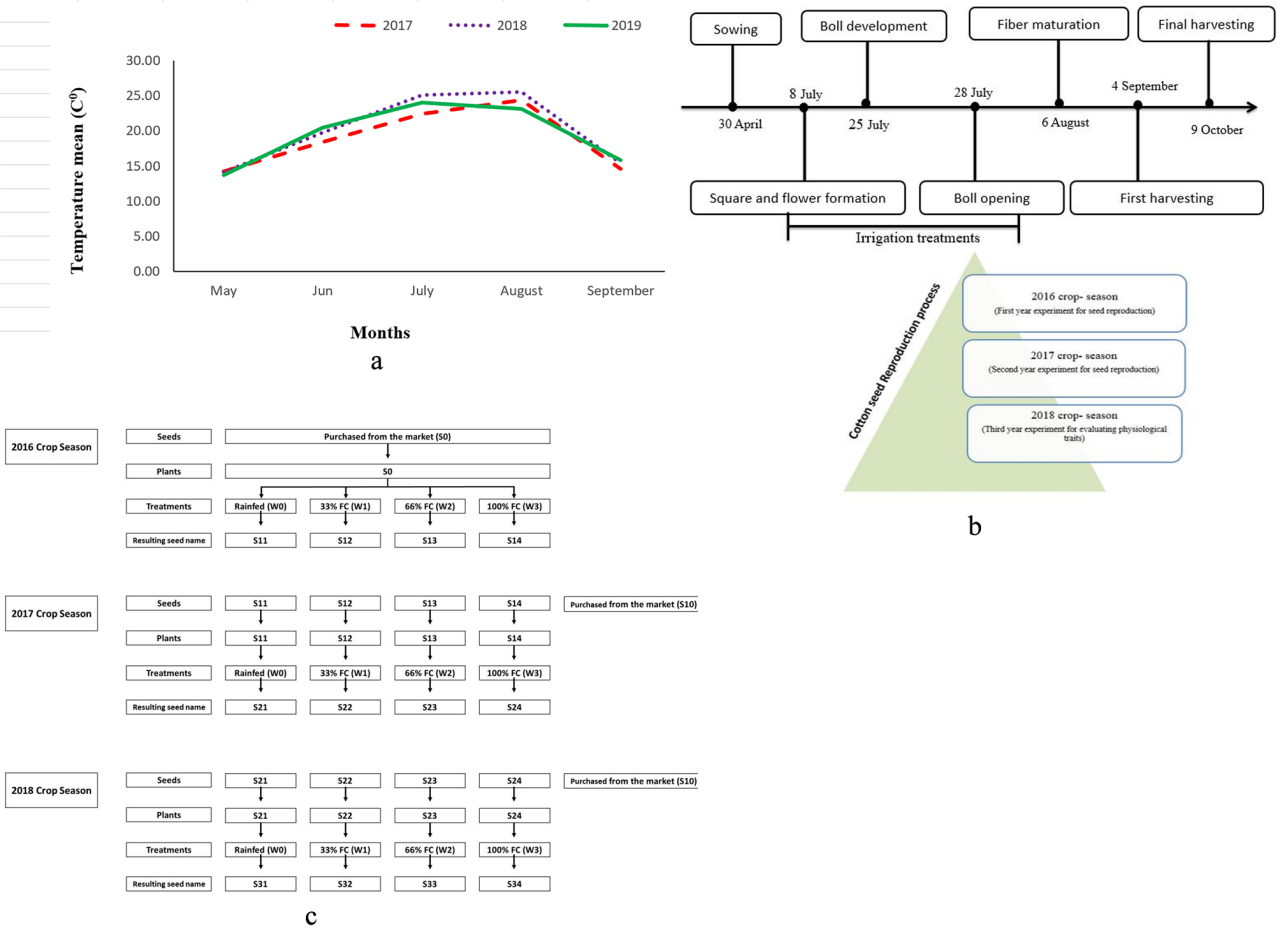
(**a**) temperature average (sowing to harvesting time) during three year, (**b**) irrigation treatment time for seed reproduction during three years and (**c**) Diagram of conducting three years of experiment (It was obtained by Correspond author)

In order to investigate this research, the experiment was conducted at Hashemabad Cotton Research Station, which was located at the southeast corner of the Caspian Sea (36° 51’ *N* latitude, 54° 16’ E longitude, and 13.3 m above the mean sea level). The mean temperature information is presented (Fig. 1a). Cotton seeds were cultured in the soil with sandy clay silt (6, 6.8, 3) texture throughout the 0.5 m soil profile. Water content at field capacity (FC) and wilting point were 28.1% and 14.1% by volume, respectively.

As shown in Fig. (1b and 1c), in the first year (2016), all experiment seeds were purchased from the market and named S0. These seeds were cultivated and were subjected to four treatments included rainfed, 33%, 66%, and 100% of field capacity (W0, W1, W2, and W3, respectively), and the resulting seeds, which were named S11, S12, S13, and S14, were collected for cultivation in the following year. In the second crop season (2017), the seeds obtained from the first year (S11, S12, S13, and S14), along with the seeds obtained from the market (S10) were subjected to water treatment similar to the first year, as a double water-stress exposure test. Therefore, the experiment in the second year included both seed treatments and irrigation conditions. The seeds obtained in the second year, known as S21, S22, S23, and S24, along with the seeds purchased from the market (S20), were treated with rainfed, 33%, 66%, and 100% of water requirement (W0, W1, W2, and W3 respectively) in the third year (2018). The seeds obtained from these treatment conditions were named as S31, S32, S33, and S34. All the experiments were conducted following a split-plot factorial design with three replications. The seeds were planted at a 20 cm distance from each other and an 80 cm distance between rows. The amount of irrigation and the time of irrigation were determined by a water-flow meter and gravimetric methods, respectively. After the production of seeds, the third-generation seeds were placed under different irrigation conditions (S31, S32, S33, and S34) in order to investigate seed and seedling traits, including germination percentage, seed vigor, germination uniformity (Gu), seed endosperm weight, seed coat weight, 100 seed weight, Starch to carbohydrate Ratio in seed, accelerated aging, cold test, growth seedling, and seedling biomass and protein concentration. Also, Glutathione reductase was planted in laboratory conditions in a factorial format based on a randomized complete block design with three replications.

### Germination percentage

50 seeds from each resulting seed were germinated between two rolled filter papers (25 × 38 cm) with 10 mL of distilled water. Each rolled paper was placed at 25 ± 2 °C in with 250 mol m^− 2s− 1^ light intensity (diurnal cycle was 8 h light and 16 h darkness). The seeds were considered to have germinated after nine days and estimated using the following equation [36]: Gmax = 100×Germinated seed number at 9th day/ Total number of seeds.

### Seed vigour index

This experiment was conducted using the ISTA rules [20] based on the Cool-Warm test. Incubated seed at optimum temperature and germination was recorded daily. Seedling lengths were measured after seven days of incubation on 50 seedlings from each replicate. For the cool test assay, 50 cotton seeds were cultivated in the standard germination method at 18 ° C for seven days. Warm test was operated in interval (16 h/20 ^0^C and 8 h/30 ^0^C) for four days. After adding the percentage of cold test with warm test, Seeds Vigour Index for each treatment was classified (Table 1).

**Table 1:** Seed Vigor index (Ista, 1985)

Classification	Cool + warm test germination percentage
Excellent	≥ 160
Good	140–159
Fair	120–139
poor	≤ 120

### Germination uniformity (Gu)

Germination uniformity was calculated using the number of germinated seeds in each day (n), the mean of germination time (t̅), and the number of days from the beginning of germination (t) using the following formula [23].

1 $$\:CUG=\frac{\sum\:n}{\sum\:\left[{\left(\stackrel{-}{t}-t\right)}^{2}\times\:n\right]}$$

### Seed endosperm weight, seed coat weight, and 100-seed weight

100 seeds, with three replicates of each sampled seed, were randomly selected and weighed. The seeds were placed in 75 ml distilled water at 40 ± 2 °C for 48 h. Then, the average of seed coats and endosperm were separated from eachother and dried at 100 °C for 24 h, and then weighed following Liu et al. [21].

### Starch to carbohydrate ratio in seed

The sampled seeds were a homogenous mixture of seeds from the bolls collected from the upper, middle, and lower parts of cotton plants, respectively. Then, the samples were kept in liquid nitrogen for biochemical analysis in a laboratory. The total carbohydrate was quantified using Phenol-Sulfuric acid and the starch content (mg g^− 1^) was determined using the phenol–sulfuric acid method [25]. To evaluate the starch content, 10 ml distilled water was added to the dried pellet. Then, Ba (OH)_2_ (0.3 N) and ZnS0_4_ (5%) were mixed with them. After centrifuging the samples (3000 rpm, 10 min), 1 ml phenol (5%) and 5 ml sulfuric acid (98%) in 2 ml were added to the supernatant. Then, the absorbance of the extract was read at a wavelength of 485 nm to determine starch content. Finally, the starch-to-carbohydrate Ratio was estimated.

### Accelerated ageing (AA) test

200 seeds from each sampled seed using an aging temperature and time combination of 43 ± 0.5 °C for 96 ± 15 h were placed on wire mesh trays in plastic boxes and 50 mL of distilled water was added to the plastic boxes [17]. After ageing, seeds per replicate were allowed to germinate on filter paper at 25 ± 2 °C in a growth chamber for eight days.

### Growth seedling test

In order to evaluate growth seedling parameters, such as root length and seedling length, 50 seeds were cultivated in an incubator at 25 ± 2 °C on filter paper [17]. After counting the germination seeds on the eighth day, for each treatment, five seedlings were separated for shoot and root length. After separating shoot and root segments, they were kept in an oven (50 °C for 48 h). Finally, they were weighted.

### Protein concentration

The protein content was determined using Bradford reagent, where bovine serum albumin was used as a protein standard. At first, after grinding 0.5 g of fresh leaf, it was homogenized in potassium phosphate buffer. The samples were centrifuged at 4 °C for 20 min at 12,000 *g*. 2.5 mL reaction solution and 100 mg Coomassie Brilliant Blue G-250 were added to the supernatant (0.02 mL). Finally, phosphoric acid 85% (w/v) was added to this solution. The concentration of solutions was read using bovine serum albumin as a standard.

### Glutathione reductase (GR)

GR activity in each treatment was measured following Foyer and Halliwell [13] method. For this assay, 0.025 mM Na-phosphate buffer (pH 7.8), 0.5 mM GSSG, 0.12 Mm NADPH Na4, and 0.1 mL extract enzyme were prepared and reached a final volume of 1 mL. NADPH oxidation was analyzed at 340 nm. One unit of GR was defined as mg^− 1^ protein·g^− 1^ FW [4].

### In silico study

In order to conduct bioinformatics analysis, Glutathione reductase (XP_016691145.2) enzymes in *Gossypium hirsutum* protein sequence are selected from NCBI databases. Protein properties, such as sequence alignment, location, ligand binding sites, protein structure and their interactions were studied using clustral Omega, Loctree 3, COACH, PDB, STRING respectively [49].

### Statistical analysis

Statistical analysis was performed using the SPSS package program version 23.0. Data was analysed by one-way ANOVA, followed by Duncan’s multiple range test (DMRT) comparison at *P* > 0.05%.

## Results and discussion

### Seed Vigor and Gu

**Fig. 2:**
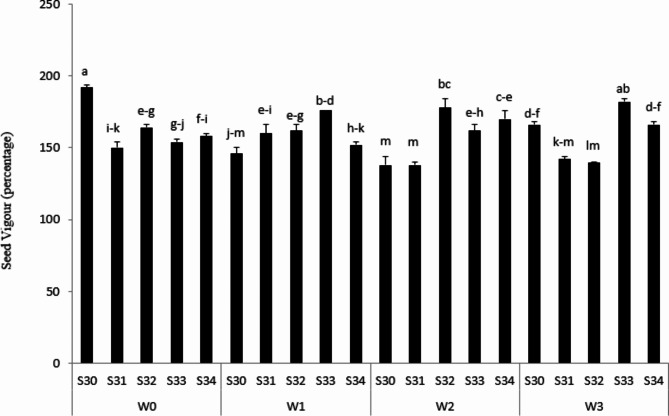
Seed Vigor (percentage) under different water treatments [Rainfed (without irrigation, 33%, 66% and 100% FC) water need] in the triple water-stress exposure

As results represented (Fig. 2), S33 under 66%FC had the best seed vigor. However, the lowest vigor was observed in S34 under rainfed conditions (Fig. 2). Seed vigor is known as a combined traits that comes from accelerated ageing seed tolerance, seed dormancy, viability, rapid germination, and seedling establishment [12]. Seed germination cannot be the best scale for plant establishment successfully, in particular in stressful conditions [32]. Therefore, evaluating seed vigor determines the potential seeds for optimum emergence percentage in the field [31]. In line with this, Wijewardana et al. [46] showed that heat stress and drought stress during seed development, in particular seed filling, cause seed vigor to reduce. Therefore, cotton seed reproduction by irrigating, 100% FC, produced weak seed vigor.

**Fig. 3:**
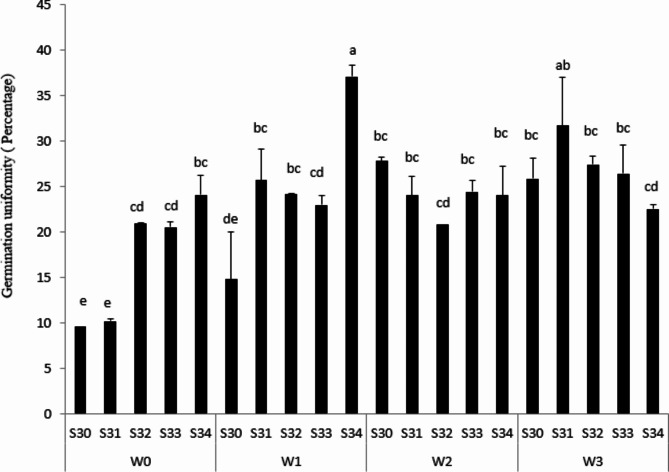
Gu (percentage) under different water treatments [Rainfed (without irrigation, 33%, 66% and 100% FC) water need] in the triple water-stress exposure

Gu results showed that S34 under 33% FC, 37.1% were the most, even though germination percentage for this treatment was 86% (Fig. 3). However, Gu in S30 under rainfed conditions was the lowest (Fig. 3). Gu, as the most important indicator for achieving suitable germination, causes the seedlings to emerge at the same time. Therefore, GU causes plant establishment to enhance [35]. These incredible approaches could be employed to select and develop drought-tolerant cotton varieties with improved root growth and seedling vigor under drought-stress conditions. Based on previous reports and studies [31], we hypothesize that there might be a strong association between early seedling vigor and root growth traits. This strong relationship might be the most important characteristic for healthy seedlings, assisting the plant facing drought stress with limited yield losses.

### Seed endosperm weight, seed coat weight and 100 seed weight

As Table 2 shows, the most seed endosperm weight was observed in S33 under rainfed conditions, while the lowest endosperm seed weight was obtained in S34 under 100%FC conditions. By increasing the amount of irrigation from 33% FC to 100%FC, endosperm seed weight was decreased (Table 2). Also, under 33% FC condition, only endosperm weight and coat weights for S32 seeds were the most (Table 2). Moreover, The highest weight of 100 seeds was in S33 under rainfed conditions. (Table 2). The average 100-seed weight in S32 under 33%FC indicated that the most seed weight was in this irrigation plot (Table 2). Based on activating memory drought stress, under 66% FC and 100% FC conditions, a decrease in 100-seed was declined by an increase in the amount of irrigation (Table 2). As Bose et al. [3] showed, growing plants under drought stress causes a change in the amount of metabolic compounds. Although the waterless priming seed process is necessary during the early stages of germination of seeds, it causes the emergence of primary root from the seed coat to prevent the emergence of the primary root from the seed coat, hence reducing seed endosperm weight [6].

**Table 2:** Physiological parameters seed under different water treatments [Rainfed (without irrigation, 33%, 66% and 100% FC) water need] in the triple water-stress exposure

W	S	Endosperm weight (g/100 seeds)	Seed coat weight (g/100 seed)	100 seed weight (g)	Seedling Biomass(g)	Seedling length (Cm )
W_0_	S_30_	8.47 ± 0.07ab	4.46 ± 0.03a-c	12.93 ± 0.14ab	0.067 ± 0.00ab	11.65 ± 0.64e-g
	S_31_	6.60 ± 0.94d-g	3.49 ± 0.03bc	10.09 ± 0.87c-g	0.061 ± 0.00a-e	12.30 ± 0.99d-e
	S_32_	7.92 ± 0.05a-c	4.24 ± 0.12a-c	12.15 ± 0.35a-c	0.059 ± 0.01a-e	9.65 ± 1.06gh
	S_33_	8.56 ± 0.0 a	4.38 ± 0.0a-c	12.94 ± 0.03ab	0.069 ± 0.00 a	12.55 ± 0.35c-e
	S_34_	5.20 ± 0.12a-c	4.10 ± 0.03a-c	12.29 ± 0.18ab	0.071 ± 0.00a	9.25 ± 1.34 h
W_1_	S_30_	7.16 ± 0.42c-f	3.72 ± 0.01bc	10.88 ± 0.45b-g	0.068 ± 0.00a	11.65 ± 0.92e-g
	S_31_	7.35 ± 0.56a-e	4.57 ± 0.10ab	11.92 ± 0.81a-e	0.064 ± 0.01a-d	11.75 ± 1.77e-g
	S_32_	8.24 ± 0.26a-c	5.32 ± 0.41a	13.55 ± 1.26 a	0.066 ± 0.00a-c	11.85 ± 1.34e-g
	S_33_	7.23 ± 1.05b-e	4.52 ± 0.33ab	11.75 ± 1.86a-e	0.054 ± 0.01c-g	10.55 ± 0.78 f-h
	S_34_	7.19 ± 0.13c-f	4.14 ± 0.21bc	11.33 ± 0.65a-f	0.068 ± 0.00a	7.10 ± 1.13i
W_2_	S_30_	7.44 ± 0.45a-d	4.52 ± 0.23a-c	11.96 ± 1.01a-d	0.047 ± 0.00 fg	12.60 ± 0.57c-e
	S_31_	6.35 ± 0.73d-g	3.72 ± 0.11bc	10.07 ± 1.00c-g	0.053 ± 0.00d-g	11.90 ± 1.27e-g
	S_32_	6.30 ± 0.43d-g	4.49 ± 0.45bc	10.78 ± 1.54b-g	0.055 ± 0.01b-f	14.85 ± 0.92a-d
	S_33_	6.15 ± 0.49 d-g	3.56 ± 0.09bc	9.71 ± 0.70d-g	0.059 ± 0.00a-e	15.20 ± 1.41ab
	S_34_	6.57 ± 0.28d-g	3.81 ± 0.10bc	10.38 ± 0.53c-g	0.062 ± 0.01 a-d	14.80 ± 0.28a-c
W_3_	S_30_	5.79 ± 0.11 g	3.72 ± 0.04bc	9.50 ± 0.21e-g	0.063 ± 0.01a-d	11.65 ± 1.48e-g
	S_31_	5.92 ± 1.18 fg	3.48 ± 0.24bc	9.40 ± 1.77e-g	0.043 ± 0.043 g	14.00 ± 0.85b-e
	S_32_	5.57 ± 0.01 g	3.48 ± 0.01bc	9.04 ± 0.03 fg	0.046 ± 0.00 fg	13.15 ± 0.49b-e
	S_33_	6.07 ± 0.73e-g	4.15 ± 0.46 a-c	10.22 ± 1.85c-g	0.046 ± 0.00 fg	14.00 ± 0.42b-e
	S_34_	5.50 ± 0.14 g	3.22 ± 0.00c	8.72 ± 0.15 g	0.049 ± 0.00e-g	16.55 ± 0.07a

### Aaccelerated ageing

**Fig. 4:**
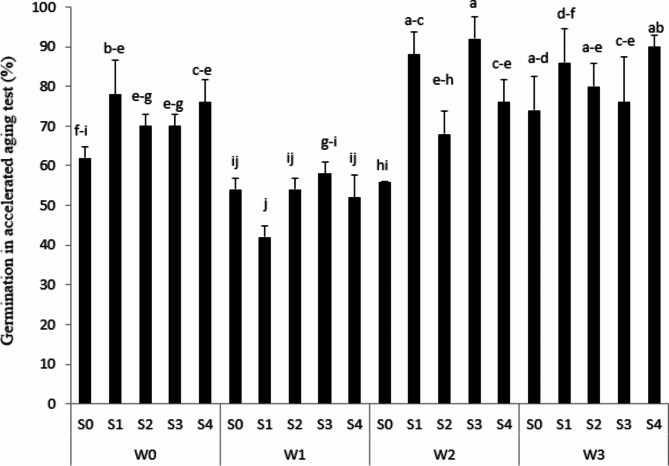
Accelerated aging seed under different water treatments [Rainfed (without irrigation, 33%, 66% and 100% FC) water need] in the triple water-stress exposure

According to the result, the lowest accelerated aging was observed in S31 under 33% FC irrigation conditions, while the most accelerated seed aging was related to S34 under 100% FC irrigation conditions (Fig. 4). As Sharma et al. [37] figured out, the accumulation of ROS, for instance ^1^O_2_, O_2_^•−^, H_2_O_2_ and OH^•^ enhanced during the process of seed aging. It is clear that storages of free radicals the cause cell membranes to be destroyed and all cell compounds such as nucleic acids, proteins, carbohydrates, and lipids hurt cell irreversibility. Figure 4 showed that S24 in the rainfed leaked the most seed storage while S20 had the lowest leakage, and that cold test is an important parameter for seed health under long period storage.

### Cold test

**Fig. 5:**
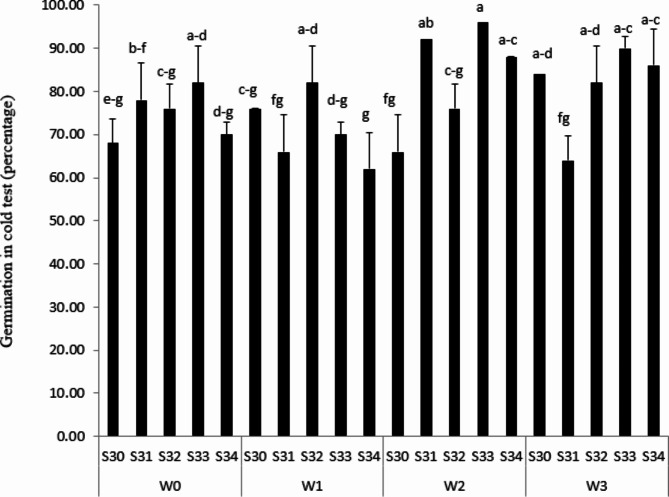
Seed in cold test (percentage) under different water treatments [Rainfed (without irrigation, 33%, 66% and 100% FC) water need] in the triple water-stress exposure

The lowest percentage of seed in the cold test was obtained at S30 in 66% FC and S31 under 33% FC irrigation conditions. However, S31 and S33, under 66% FC irrigation conditions with the most percentage, are able to have the best seed vigor for not only planting at the first sowing date but also for being more tolerant against low temperature of soil (Fig. 5). As reported by Filho [11], cold test and accelerated aging are known as an indicator for evaluating seed tolerance to stress.

### Seedling biomass and seedling length

Results represented that S31 and S32 under rainfed and 33% FC conditions had the most seedling biomass (Table 2), though they had the lowest seedling length (Table 2). As Saha et al. [33] reported, the accumulation of some osmolites under drought stress causes the seeds to be more tolerant. Moreover, S33 and S34 under 66% FC and 100% FC had the highest dry weight, respectively. As Faghani et al. [9] observed, stomatal conductance was declined in the leaves of S33 and S34 under 100% FC irrigation conditions. S30 under 66% FC had the most seedling length in comparison to other seed treatments even though it had the lowest seedling biomass (Table 2). Under the rainfed condition, S33 had the most seedling biomass (Table 2). It is obvious that several plant species are equipped with drought stress memory on the physiological and biochemical levels in order to minimize water loss, regulate ROS homeostasis, and change photosynthetic rates through changing phytohormone contents or in biomass [28]. Also, as many studies revealed, drought stress caused a decrease in germination, seedling growth, root and shoot dry weight, cleoptile length and vegetative growth (32, 22), which can be influenced by the loss of turgor and followed by limitation in the process of cell growth [42]. However, in the present study, when the plant was grown from seed sources obtained from drought conditions, it had the ability to overcome the negative effects of drought and it can be considered to be related to a phenomenon called ‘stress memory’.

### Starch to carbohydrate ratio in seed

**Fig. 6:**
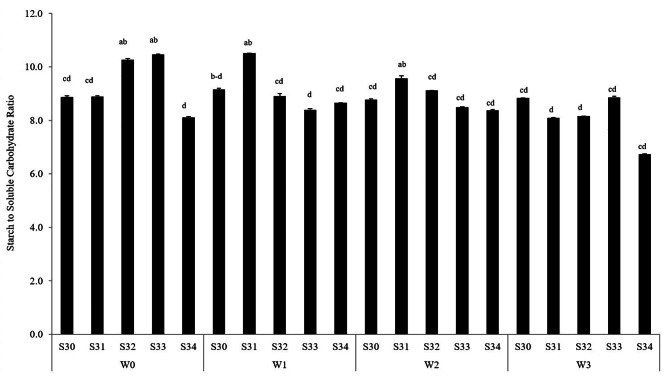
Starch to carbohydrate ratio in seed under different water treatments [Rainfed (without irrigation, 33%, 66% and 100% FC) water need] in the triple water-stress exposure

As results indicated (Fig. 6), in rainfed conditions, S33 had the most starch content to soluble carbohydrate ratio. According to memory drought stress effects, the ratio of starch content to soluble carbohydrates causes to increase seed weight. As Hlahla et al. [19] results showed, the significant increase in the starch content in the seed of drought-tolerant cultivars is strongly related to the fundamental roles of starch for evaluating drought stress tolerance. Moreover, under both 33% and 66% FC conditions, due to acting memory drought stress, the ratio of starch to carbohydrate soluble in the seeds of S31, 10.3% and 14.8% were more than that in the S30 seeds. On the other hand, if the field has enough water sources for irrigating under high irrigation conditions, S30 will accumulate the most starch to soluble carbohydrate in the seed (Fig. 6). Furthermore, the glucose storage in S34 was more than starch by irrigation 100% FC. It means that S34 is sensitive to high irrigation (100% FC). It is clear that during drought stress, starch should be degraded in order to replenish glucose needs in the plant cell [24]. Hence, following this approach can lead to a balance in the content of glucose storage so that the photosynthesis process under the drought stress condition is protected [1]. As Hlahla et al. [19] concluded, the accumulation of starch in seeds directly correlates with seed mass per plant.

### Protein concentration and glutathione reductase (GR)

**Fig. 7:**
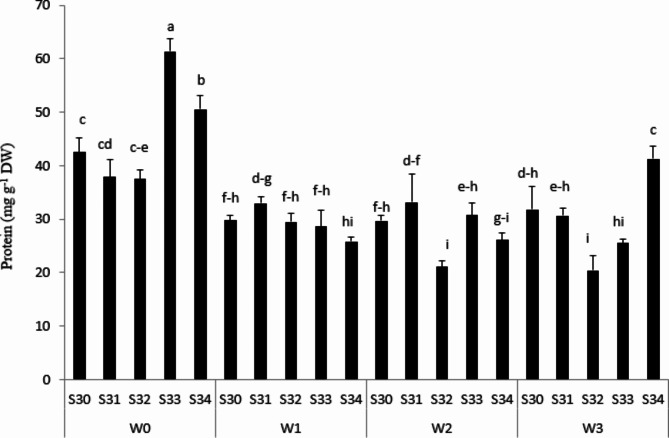
Protein concentration in seed under different water treatments [Rainfed (without irrigation, 33%, 66% and 100% FC) water need] in the triple water-stress exposure

Although the most protein storage was observed in S33 under rainfed, the lowest protein concentration was obtained in S32 under 66%FC irrigation conditions (Fig. 7). It can be concluded that the increase of 100-seed weight and seed dry weight in S33 in the rainfed condition derived from high protein storage in S33 seed (Table 2). Quite related to this, Rakszegi et al. [30] showed that severe drought causes the protein concentration of seeds to increase, while decreasing in protein storage of seed is known as a tolerant index.

**Fig. 8:**
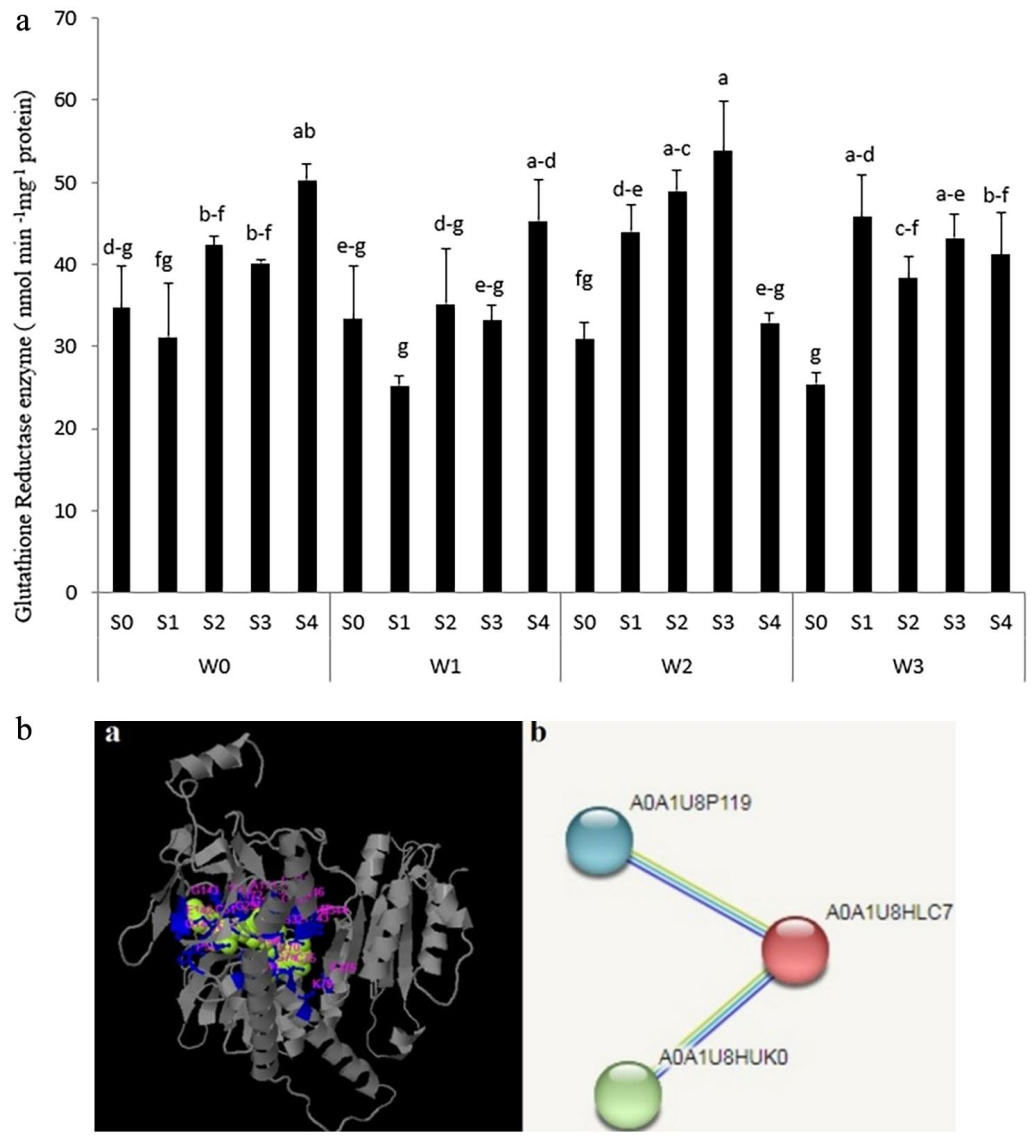
(**a**) Glutation activity enzyme in seed under different water treatments [Rainfed (without irrigation, 33%, 66% and 100% FC) water need] in the triple water-stress exposure. (**b**) Protein structure of Glutation Reductase (***a***) The 3D structure (***b***) proteins interactions of Glutation Reductase. GR (A0A1U8HLC7), (A0A1U8HUK0)and Glutathione transferase (A0A1U8P119)

GR activity in the seeds of S33 under 66% FC condition was observed to be the highest while the amount of this enzyme was increased in S34 under rainfed conditions. Based on memory drought stress activation, with an increase in the level of irrigation S34 seeds, as a genetic resource, is sensitive to o tolerate rainfed conditions. On the other hand, S31 under the rainfed and 33% FC conditions had the lowest GR (Fig. 8a). Then, activating memory stress in S31 could be more tolerant against drought stress due to having low GR activity. It is obvious that the accumulation of the radical form of oxygen was too low to activate GR. It is clear that S31 seeds under 66% FC and 100% FC conditions had 74.3% and 81.5% glutathione activity, respectively, which was more than that in 33% FC irrigation conditions. Therefore, perceiving drought stress signals and increasing the radical form of Oxygen and free radicals, glutathione enzyme is activated more to export damaged effects of drought stress. Consequently, Glutathione, as a main scavenger of O._2_, H_2_O_2_, and OH·, can counteract the inhibitory effects of ROS that are induced by oxidative stress and cause cells to have the normal status in this condition [27]. Szalai et al. [41] found that Glutathione, as a substrate or co-factor for a number of biochemical reactions, interacts with hormones and redox molecules. So, it plays a crucial role in stress-induced signal transduction to remediate drought aspects.

### In-silico analysis of GR

Bioinformatic analysis of protein structure GR (XP_016691145.2) in cotton indicated that GR (molecular weight 60 kDa, oxidoreductase family) contains a Redox-active site and NADPH binding domain. The second structure of GR consists of 28% alpha-helix and 14% Beta sheet. In this sequence, Cystein amino acid was highly conserved in the catalytic site of the enzyme. In-silico analysis showed that XGXGXA motifes and arginine amino acid were in the NADPH binding domain. Also, active sites in GR were determined in 51, 52, 33, 31, 32, 28, 29,172, 171, 143, 141, 79, 75, 74, 73, 72, 70, 69, 68,54,53, 375, 346, 344, 343, 341, 335, 334, 298, 295, 211, 190, 173 positions (Fig. 8b). Glutathione disulfide (GSSG) and Nicotinamid-Adenine dinucleotide (NAD) were considered GR subestrate. The study of intercellular GR determined that GR is in cytosol and chloroplast of cotton (Table 2). It is clear that the lowest and highest affinity values between substrate and enzyme were approximately − 5 to − 5.8 kcal/mol, respectively (Table 2). Generally, the results proved that GR interacted with GST(Glutation –S-transferase).

Protein sequence GR is comprised of two cysteine residues (Redox-active site), a dimerization domain, and an NADPH binding domain that showed high conservation in different species [22]. Investigations showed that GR sequences have 10–16 exsons and gene expression detected in leaves, roots, pheloem, and buds of plants. Based on Phylogenetic evaluations, this protein is divided into clades GR (I and II). Clade I codes proteins that are related to cytosol [47]. GR activity in chloroplasts plays a vital role in chloroplast protection against oxidative stress and is available for reduced glutathione. Also, affecting GSH/GSSG ratio, GR can balance cellular redox in cytosol and mitochondria [50]. The cellular location of GR has been reported in cytoplasm, chloroplast, and mitochondria in different species. This issue is rooted in the duplication process during evolution and protection of cellular balancing [47]. Abiotic stress effects such as the structure of the enzyme, and protein denaturation influence its function by decreasing tendency of the enzyme and the substrate. Then, abiotic stress, can modify the phosphoralation and acetylation of GR and hence the stability of the enzyme decreases [22].

The results of the in-silico analysis determined that GR interacted with GST [43]. Although GR and GST have different functions, they can collabrate for balancing cellular redox and detoxification processes [8]. GST plays an important role in the detoxification of toxic compounds and the high activity of this enzyme causes harmful aspects to cells; consequently, it leads to high tolerance to exposure to abiotic stress [14]. The interaction of GR and GST is conducted through preparing GSH and its usage. Finally, GR protects GST function indirectly. Moreover, GST is able to catalyze the reduction of GSSG. This reaction is known as a subsitution pathway. Therefore, it helps to stabilize optimum GSH/GSSG ratio in cells [29].

## Conclusion

In General, memory drought stress information of S31, such as reducing accelerates aging seed percentage, evaluating GR activity, the best percentage of Gu and seed vigor showed that this pattern could be recommended for these fields. Therefore, S31 can be introduced for early sowing date and cold regions by supplying 33% of the required water because of having the highest percentage of germination seeds in cold test analysis. It should be noted that, for rainfed fields, the seeds that were reproduced under third 66% FC water exposure conditions had the highest shoot weight and shoot length for seedling, protein content, and starch to carbohydrate ratio in seed and seed biomass. Then, seeds reproduction with 33% and 66% FC$\:\frac{2}{3}$will produce more tolerant seeds for the fields without rainfall and water sources, which can be a response to stress memory. In-silicon analysis revealed that GR was located in cytosol and chloroplast and interacted with GST. Generally, the effective role of GR in controlling oxidative stress and acclimation with environmental conditions can be derived from memorial stress during the plant development stage.

## Data Availability

Data is provided within the manuscript or supplementary information files.
